# Pregnancy-Associated Plasma Protein A (PAPP-A) as a Predictor of Third Trimester Obesity: Insights from the CRIOBES Project

**DOI:** 10.3390/pathophysiology31040046

**Published:** 2024-11-15

**Authors:** Inmaculada Gabaldón-Rodríguez, Carmen de Francisco-Montero, Inmaculada Menéndez-Moreno, Álvaro Balongo-Molina, Ana Isabel Gómez-Lorenzo, Rubén Rodríguez-García, Ángel Vilches-Arenas, Manuel Ortega-Calvo

**Affiliations:** 1 Andalusian Health Service, Primary Care Seville District, 41004 Seville, Spain; mariai.gabaldon.sspa@juntadeandalucia.es (I.G.-R.); carmendfm@gmail.com (C.d.F.-M.); inmamenenm@gmail.com (I.M.-M.); colimbo16@gmail.com (Á.B.-M.); anaisabelgomezlorenzo@gmail.com (A.I.G.-L.); rubrodgar95@gmail.com (R.R.-G.); 2 Preventive Medicine Department, University of Seville, 41009 Seville, Spain; ava@us.es

**Keywords:** predictive models, obesity, placental hormones, pregnancy, primary care

## Abstract

**Introduction:** Our objective in this article was to develop a predictive model for obesity in the third trimester of pregnancy using the plasma and clinical biomarkers that are managed within the Chromosomopathies Programme in the Andalusian Public Healthcare System. **Methods:** The epidemiological design was observational, of the unmatched case–control type. The geographical environment was the Seville Primary Healthcare District (DSAP Sevilla). The information was collected between 2011 and 2021. The reference cohort consisted of women who had carried a pregnancy to term. The variables and biomarkers studied correspond to those managed within the primary-care Pregnancy Integrated Care Pathway (ICP). Unconditional binary logistic regression (BLR) models were created, with the outcome variable being whether or not the women were obese in their third trimester of pregnancy. **Results**: A total of 423 controls and 104 cases of obesity were obtained for women in their third trimester who had not been obese in their first trimester. The average age for the sample group (P50) was 34 years old. The final, most parsimonious model included the variables PAPP-A (*p* = 0.074), beta-hCG (*p* = 0.1631), and systolic blood pressure (SBP) (*p* = 0.085). ROC curve = 0.75 (C.I. at 95%: 0.63–0.86). **Discussion**: The results of this research can only be extrapolated to primary care and to pregnancies with no complications. PAPP-A has been shown in our research to be a significant predictor of obesity risk in the third trimester of pregnancies with no complications (OR = 0.53; C.I. at 95%: 0.39–0.66; *p* = 0.04 in the single-variant study; OR = 0.58; C.I. at 95%: 0.29–0.93; *p* = 0.074 in the multi-variant analysis). This predictive capacity is further enhanced from an operational perspective by beta-hCG and 12-week SBP.

## 1. Introduction

Obesity is a risk factor for disorders such as diabetes mellitus [1], cardiovascular disease [2], neoplasms [3], and premature death [4]. Obesity may also affect the clinical development of many chronic conditions. Obesity during pregnancy has been linked to an increase in births via caesarean section [5].

The BMI or Quetelet´s index is one of the most used anthropometric measurements in daily clinical practice, and is calculated by dividing a person’s body weight, expressed in kilograms, by their height, expressed in meters and squared (BMI = weight (kg)/height (m^2^)) [6].

Pregnancy-associated plasma protein A (PAPP-A) is a zinc metalloproteinase that was identified in 1974 as a placenta-derived protein in pregnant women [7]. PAPP-A was subsequently shown to be a useful marker for Down syndrome during pregnancy. Although the placenta is the main source of PAPP-A, several studies have also reported its existence in tissues such as the testis, kidney, and colon, and its expression has also been found during injury repair and remodeling processes, during skin healing, and in vascular smooth muscle cells [8,9].

Furthermore, the beta portion of the human chorionic gonadotropin hormone (β-hCG), which was discovered in the urine of pregnant women by Aschein and Zondek in 1927 [10], is a glycoprotein produced by the syncytiotrophoblast. The primary function of β-hCG is to produce hormones such as progesterone and estrogen, which keep the endometrium growing and producing nutrients. If the corpus luteum is removed before the 7th week of pregnancy, this almost always leads to a termination. In clinical practice, it is also used for the diagnosis of trophoblastic and non-trophoblastic tumors [11,12].

Third-trimester obesity is associated with risks for both the mother and the fetus [5,8].

Our objective in this article was to develop a predictive model for obesity in the third trimester of pregnancy using the plasma and clinical biomarkers that are managed within the Chromosomopathies Programme in the Andalusian Public Healthcare System.

## 2. Material and Methods

The epidemiological design was observational, of the unmatched case–control type [13,14]. The geographical environment was the Seville Primary Healthcare District (DSAP Sevilla). The information was collected between 2011 and 2021. The reference cohort consisted of women who had carried a pregnancy to term within the Seville Primary Healthcare District.

### 2.1. Inclusion and Exclusion Criteria

The study included pregnant women who had accessed the Pregnancy Integrated Care Pathway (ICP) at one of the sampled healthcare centers. If, during the course of the pregnancy, they presented with criteria for gestational diabetes, they remained in the study. Participants were required to be aware of and to have signed the informed consent form and the information sheet for our research project.

A woman was considered a “case” if she met WHO criteria for obesity in the third trimester without being classed as obese in the first trimester. A woman was considered a “control” if she did not meet the WHO criteria for obesity in the third trimester without being classed as obese in the first trimester. Women were excluded if they had suffered from any significant complications in relation to their pregnancy, such as eclampsia, medium–severe arterial hypertension, or miscarriage. There were no age limits. If only one visit was made to the Pregnancy ICP, this was considered an exclusion criterion.

### 2.2. Sample

Information was collected ambispectively from a total of seven health centers in the Seville district (“Las Palmeritas”, “Amante Laffón”, “Ronda Histórica”, “El Greco”, “Esperanza Macarena”, “Montequinto”, and “El Cachorro”). The information was collected from the Diraya Programme and the SiPACAC application [15,16].

### 2.3. Sample Size Calculation

Based on the objectives, a sample size calculation was performed with a case–control design using the IMIM GRANMO program. Accepting an alpha risk of 0.05 and a beta risk of 0.2 (80% power) in a bilateral contrast, 121 cases and 484 controls were required to detect a minimum odds ratio (OR) of 0.5. The rate of exposure in the control group was assumed to be 0.80. A loss to follow-up rate of 0% was estimated. The POISSON approximation was used [17]. We also took into account the criteria established by Concato, Perduzzi et al. [18,19,20].

### 2.4. Variables

The variables and biomarkers studied correspond to those managed within the primary-care Pregnancy Integrated Care Pathway (ICP).

1. Obesity in the third trimester.

Conceptual definition: being or not being classified as obese at 28 weeks (binary category).

Working definition: variable measurement: presenting with a BMI of over 30.00 kg/m^2^ (dependent variable).

2. Plasma PAPP-A.

Conceptual definition: plasma PAPP-A in chromosomal screening (continuous quantitative).

Working definition: plasma PAPP-A level recorded in chromosomal screening. Collected from the Sipacac application. Unit: mUI/mL.

3. Beta-hCG.

Conceptual definition: Beta-hCG in the chromosomal screening (continuous quantitative variable).

Working definition: plasma Beta-hCG level recorded in chromosomal screening. Collected from the Sipacac application. Unit: ng/mL.

4. BMI at 12 weeks.

Conceptual definition: BMI recorded during Pregnancy ICP appointment at 12 weeks.

Working definition: BMI value recorded during Pregnancy ICP at 12 weeks of pregnancy (continuous quantitative variable). Unit: kg/m^2^.

5. Basal glucose at 12 weeks of pregnancy.

Conceptual definition: Basal glucose level at the first trimester of pregnancy as an expression of carbohydrate metabolism (continuous quantitative variable).

Working definition: basal blood sugar level at the first Pregnancy ICP appointment. Unit: mg/dL.

6. Basal glucose at 28 weeks of pregnancy.

Conceptual definition: basal glucose level at the second trimester of pregnancy as an expression of carbohydrate metabolism (continuous quantitative variable).

Working definition: basal blood sugar level at Pregnancy ICP appointment during second trimester (continuous quantitative variable). Unit: mg/dL.

7. BMI at 28 weeks of pregnancy.

Conceptual definition: BMI recorded during Pregnancy ICP appointment at 28 weeks.

Working definition: BMI value recorded during Pregnancy ICP appointment at 28 weeks of pregnancy (continuous quantitative variable). Unit: kg/m^2^.

8. SBP (systolic blood pressure) at 12 weeks of pregnancy.

Conceptual definition: SBP recorded during Pregnancy ICP appointment at 12 weeks.

Working definition: SBP value recorded during the Pregnancy ICP at 12 weeks of pregnancy (continuous quantitative variable). At rest after sitting down for five minutes. Unit: mm Hg.

9. DBP (diastolic blood pressure) at 12 weeks of pregnancy.

Conceptual definition: DBP recorded during Pregnancy ICP appointment at 12 weeks.

Working definition: DBP value recorded during Pregnancy ICP at 12 weeks of pregnancy (continuous quantitative variable). At rest after sitting down for five minutes. Unit: mm Hg.

10. DBP at 28 weeks of pregnancy.

Conceptual definition: DBP recorded during Pregnancy ICP appointment at 28 weeks.

Working definition: DBP value recorded during Pregnancy ICP at 28 weeks of pregnancy (continuous quantitative variable). At rest after sitting down for five minutes. Unit: mm Hg.

11. Free T4 at 12 weeks of pregnancy.

Conceptual definition: free T4 recorded during Pregnancy ICP appointment at 12 weeks.

Working definition: free T4 level recorded during Pregnancy ICP at 12 weeks of pregnancy (continuous quantitative variable). Unit: ng/dL.

12. TSH at 12 weeks of pregnancy.

Conceptual definition: TSH recorded during Pregnancy ICP appointment at 12 weeks.

Working definition: TSH level recorded during Pregnancy ICP at 12 weeks of pregnancy (continuous quantitative variable). Unit: µUI/mL.

13. Free T4 at 28 weeks of pregnancy.

Conceptual definition: free T4 recorded during Pregnancy ICP appointment at 28 weeks.

Working definition: free T4 level recorded during Pregnancy ICP at 28 weeks of pregnancy (continuous quantitative variable). Unit: ng/dL.

14. TSH at 28 weeks of pregnancy.

Conceptual definition: TSH recorded during Pregnancy ICP appointment at 28 weeks.

Working definition: TSH level recorded during Pregnancy ICP at 28 weeks of pregnancy (continuous quantitative variable). Unit: µUI/mL.

We do not consider body composition variables [21]. Resistance to insulin could not be analyzed either, because this variable is not included in the Maternal ICP for primary healthcare.

### 2.5. Ethical Considerations

The Local Research Ethics Committee approved the two projects (CRIOBES-CRIVENTOS) on which this article is based in 2012 and 2020 [22].

### 2.6. Analytical Phase

Unconditional binary logistic regression (BLR) models were created [23,24,25,26], with the outcome variable being **whether or not the women were obese in their third trimester of pregnancy**. To decide which model was best, we took into account the stepwise backward–forward analysis, the ROC curve surface generated by each of them [27], and the accuracy plots for each of the models. We also looked for interaction variables. The statistical analysis was carried out using the R package version 3.5.3 (“Great Truth”) [28] and the R Commander interface [29] version 2.5–2 (plug-in KMggplot2, ROC, NMBU, Optim Classifier, Pca Robust and Plot by Groups) [30,31]. The ROC curves were obtained with the ROC plug-in, and the confidence intervals were obtained using 2000 bootstrap samples [32,33].

## 3. Results

Table 1, Table 2 and Table 3 show most of the descriptive results of our research project.

The dependent variable of this article is based on the results obtained in the second trimester (obese = 104 records (CASES); non-obese = 214 + 209 records) (CONTROLS).

**Figure 1:**
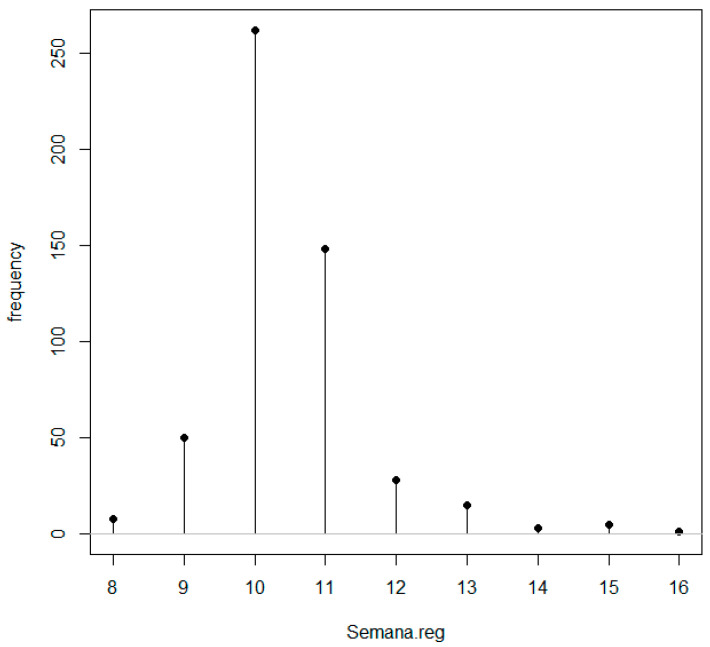
Week of pregnancy when screening was performed (discrete quantitative variable). Measured according to information from DIRAYA or the chromosomal screening. A total of 409 records were taken between the 10th and 11th week of pregnancy (78.8%).

Figure 1 shows that the majority of measurements at the first trimester of pregnancy were taken between the tenth and eleventh week.

Single-variant analysis with unconditional binary logistic regression.

Multi-variant model with three predictors on the outcome variable of women who were obese/not obese at their third trimester of pregnancy but were NOT obese at their First Trimester of Pregnancy. Number of records with complete information = 290.

**Figure 2:**
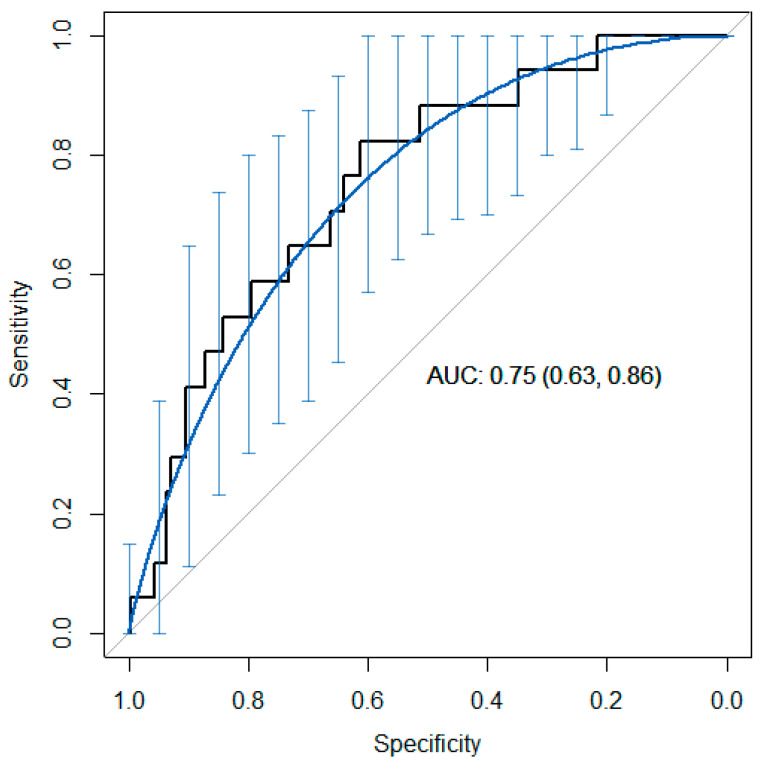
Model ROC curve from Table 5 (most parsimonious).

**Figure 3:**
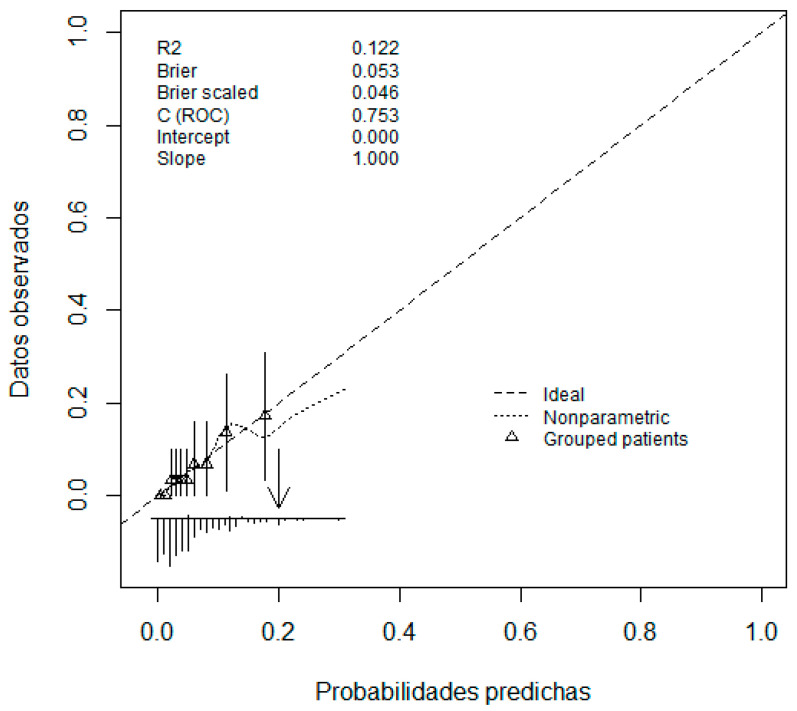
Calibration graph of most parsimonious model shown in Table 5 (three predictor variables; *x*-axis predicted probabilities; *y*-axis observed data).

The univariate analysis of each of the variables is shown in Table 4 and Table 5. The most parsimonious multi-variant model was built via binary logistic regression (BLR). Figure 2 and Figure 3 show the graphical results obtained from the final, most parsimonious multi-variant model (ROC curve and calibration graph).

## 4. Discussion

In this article, we attempted to create a prediction model for obesity in the third trimester of pregnancy for women who were not obese in their first trimester. The hypothesis of the study is that PAPPA could be a clinical marker of obesity in the third trimester independent of diet in uncomplicated pregnancies.

The first objection is the amount of lost information that could not be accessed (Table 3). At no point were we willing to perform data imputations [34,35]. We had to reject over a hundred records due to lack of information in DIRAYA and SiPACAC. The retrospective aspect of this ambispective study is undoubtedly another weakness [36]. No matching took place.

No specific dietary interventions were carried out. The ICP recommends a Mediterranean diet (MD) as the healthiest option [37,38], but the women were always free to eat what they wanted, as the majority of pregnancies studied were free from complications. Patients presenting criteria for gestational diabetes received specific dietary advice. This project was carried out and analyzed in primary care, meaning that it is a community study. Its results are difficult to extrapolate to a hospital environment [39]. Huang et al. recently published observational results showing higher PAPP-A values in HBV-positive women [40]. We did not consider this variable.

The first advantage is the sample size achieved. After a phase in which we curated and adjusted the data package [41], we ended with a total number of 572 records. Despite the amount of information lost, this sample size gave the BLR models an adequate level of statistical power.

We believe that the longitudinal observation time and the number of health centers studied make the sample highly representative. In accordance with what was posited by Silva-Ayçaguer, we believe that the term “representative sample” can never have a standardized meaning [42,43]. The prospective part of this ambispective study is undoubtedly a strength [44].

We conducted an in-depth analysis of the linear correlations between the predictors and the outcome variable in its continuous form (BMI T2), which allowed us to select a BLR model with low internal collinearity [45,46]. The BMI at T1 was not included in the final model as it demonstrated very high levels of collinearity.

We sincerely believe that, with this study, we are increasing the efficiency [47,48,49] of the Primary Care Pregnancy ICP, because by just using the information contained within, without spending a single extra euro, we have developed an instrument for predicting obesity in the third trimester of pregnancy. The time spent recording the information in the data package was outside of the health center’s working day. This was the most significant investment.

We chose the most parsimonious multi-variant BLR model possible, with the lowest level of internal collinearity (Figure 2; Table 5) [50].

There is a comment of a qualitative nature that should be made about this essentially quantitative study [51,52]. We would like to highlight the highly positive response we received from most of the pregnant women when they were informed that they were taking part in a Primary Care Observational Research Project. The initial reaction of astonishment was generally followed by statements of gratitude, infusing most of the interviews with a positive feeling [53].

This article was produced as a result of two clinical research projects carried out in primary care [54,55,56]: CRIVENTOS [57] and CRIOBES. The first project began over 10 years ago when we were trying to elucidate predictor variables for Gestational Diabetes, and the second project, which serves as the framework for this study, was focused on obesity in the third trimester of pregnancy as a final variable.

The size of our total raw sample was 572 records (Table 3). With this sample size (N), we had a sufficient margin to respect the maximum of ten events per variable in the analysis with BLR [58,59]. Although other researchers have since researched this matter, they have not looked at it in much depth [60,61,62].

Chen et al. [63] recently studied weight gain in pregnant women who have undergone in vitro fertilization, but they did not obtain any significant results with respect to PAPP-A.

Although we worked with logarithmic transformations during the analysis phase, with the final model, we chose to leave the natural distribution as it was for the PAPP-A variable and Beta-hCG [64]. Figure 1 shows that the vast majority of the extractions for the records were taken during the tenth and eleventh weeks of pregnancy.

The average age of the pregnant women was 34 years old (Table 3). The 50th percentile is the best estimator of central tendency in time variables, so we accept that this result is illustrative of the reproductive age of women in Seville in recent years. It is important to remember that the included pregnancies may or may not have been first pregnancies, but only one was included per woman. It should also be noted that, due to inclusion and exclusion criteria, the vast majority of pregnancies were free from complications.

The PAPP-A and Beta-hCG biomarkers did not correlate with the weight gain or BMI gain variables using simple linear regression models. We can therefore confirm that there was no internal collinearity.

One very interesting observation we made was the lack of significance and zero-predictive contribution of TSH at 12 weeks on obesity status in the third trimester. However, the literature contains data that would contradict this observation. Collares et al. found that elevated TSH levels during the first trimester of pregnancy were associated with the development of obesity during the third trimester [65]. Svare et al. also reached the same conclusions a few years earlier [66]. Wei et al., on the other hand, demonstrated a connection between fasting plasma glucose and fetal weight in women with gestational diabetes [67].

We took into account the Brier index in the calibration graph, although certain authors question its validity [68]. In the three-variable model, the Brier index is 0.053 (a perfect model would have a Brier index of zero) and the scaled Brier index is 0.046 (the Brier index ranges from 0 to 1; the higher the value, the better the predictions). We chose a model with the lowest number of variables (Table 5), eliminating those with the highest collinearity (BMI T1 and extraction week), following Occam’s Principle of Parsimony [69] and Ludwig Wittgenstein’s logical propositions (“Simplex sigillum veri”—Simplicity is the sign of truth) [70]. The fundamental arguments for selecting the three-variable model were control of collinearity and parsimony. Although a backward–forward stepwise analysis of the more parsimonious model left the PAPP-A variable as it was, it did so at the expense of a loss in the area under the ROC curve (0.67 versus 0.75 for the model with all three variables) (Figure 2). Although we did look for models with interaction variables, no significant or operational results were obtained. We believe that this work provides an additional instrument (PAPP-A) as a predictor of obesity risk in pregnancies which are not complicated from an obstetric point of view.

In summary, our research has shown PAPP-A to be a significant predictor of third-trimester obesity risk in pregnancies without complications (OR = 0.53; C.I. at 95%: 0.39–0.66; *p* = 0.04 in the single-variant study (Table 4); OR = 0.58; C.I. at 95%: 0.29–0.93; *p* = 0.074 in the multi-variant analysis) (Table 5). This predictive capacity is further enhanced from an operational perspective by beta-hCG and 12-week SBP.

## Figures and Tables

**Table 1:** Frequencies and percentages of the weights of the entire sample of pregnant women in their first trimester according to WHO criteria.

	Number of Women	Percentage	C.I. of the Percentage at 95%
Normal weight	353	63.38	59.4–67.4
Overweight	131	23.52	18.3–25.9
Obese	73	13.11	10.1–15.8

**Table 2:** Frequencies and percentages of the weights of the entire sample of pregnant women in their second trimester according to WHO criteria.

	Number of Women	Percentage	C.I. of the Percentage at 95%
Normal weight	214	40.61	36.6–45.0
Overweight	209	39.66	35.5–43.8
Obese	104	19.73	16.5–23.2

**Table 3:** Continuous variables. Descriptive statistics.

	Arithmetic Mean	Median	Standard Deviation	Q1	Q3	*n*	Lost Values
Age at pregnancy (in years)	33.45	34	4.79	30	37	572	3
PAPP-A mU/mL.	2.00	1.39	2.91	0.84	2.22	506	69
Ln PAPP-A	0.33	0.32	0.79	−0.17	0.79	506	69
Beta-hCG	68.25	56.29	48.30	34.25	91.3	545	30
Week of pregnancy at screening	10.43	10.00	1.06	10.0	11.0	521	54
BMI T1	24.62	23.51	4.78	21.3	26.4	558	17
BMI T2	26.84	25.94	4.84	23.5	29.1	528	47
Basal Glucose T1	80.31	80	9.73	74	85	547	28
Basal Glucose T2	75.34	74	12.15	68	81	535	40
SBP T1 mmHg	108.27	110	12.95	100	117	384	191
DBP T1 mmHg	68.55	69	8.58	61	74	384	191
SBP T2 mmHg	106.99	107	11.32	100	115	378	197
DBP T2 mmHg	66.99	67	8.67	60	73	378	197
TSH T1	2.08	1.75	2.08	1.09	2.55	374	201
TSH T2	2.46	2.24	2.46	1.52	2.97	177	398
Free T4 T1	1.21	1.20	0.29	1.08	1.31	134	441
Free T4 T2	0.96	0.96	0.14	0.86	1.05	156	419
BMI gain	2.16	2.06	1.53	1.19	2.97	526	49
Weight gain (kg)	5.827	5.5	4.332	−5.6	8	537	38

**Table 4:** Outcome variable: women who are obese/not obese at their third trimester of pregnancy but were NOT obese at their first trimester of pregnancy. Number of records = 290.

	Estimated Coefficient	Odds Ratio	Significance
Blood sugar T1	0.010	1.010	0.68
PAPP-A	−0.063	0.532	0.044
Beta-hCG	−0.012	0.987	0.10
SBP T1	0.045	1.047	0.037
Age of the mother	0.008	1.008	0.86

**Table 5:** Most parsimonious multi-variant model.

	Estimated Coefficient	C.I. of the Coefficient at 95%	Odds Ratio	C.I. of Odds Ratio	Significance
PAPP-A	−0.532367	−1.22; −0.065	0.587	0.294; 0.936	0.0744
Beta-hCG	−0.010515	−0.027; 0.002	0.989	0.972; 1.002	0.1631
SBP T1	0.037	−0.004; 0.081	1.038	0.995; 1.085	0.085

## Data Availability

The data are available to anyone who requests it.
